# Embryonic anti-aging niche

**DOI:** 10.18632/aging.100333

**Published:** 2011-05-31

**Authors:** Irina M. Conboy, Hanadie Yousef, Michael J. Conboy

**Affiliations:** ^1^ Department of Bioengineering and QB3 Institute, UC Berkeley, Berkeley, CA 94720-3220, USA; ^2^ Department of Molecular and Cellular Biology, UC Berkeley, Berkeley, CA 94720-3200, USA

**Keywords:** stem cell aging, regeneration, niche, senescent, cell cycle, Notch, TGF-β, MAPK, muscle, hESC

## Abstract

Although functional organ stem cells persist in the old, tissue damage invariably overwhelms tissue repair, ultimately causing the demise of an organism. The poor performance of stem cells in an aged organ, such as skeletal muscle, is caused by the changes in regulatory pathways such as Notch, MAPK and TGF-β, where old differentiated tissue actually inhibits its own regeneration. This perspective analyzes the current literature on regulation of organ stem cells by their young versus old niches and suggests that determinants of healthy and prolonged life might be under a combinatorial control of cell cycle check point proteins and mitogens, which need to be tightly balanced in order to promote tissue regeneration without tumor formation. While responses of adult stem cells are regulated extrinsically and age-specifically, we put forward experimental evidence suggesting that embryonic cells have an intrinsic youthful barrier to aging and produce soluble pro-regenerative proteins that signal the MAPK pathway for rejuvenating myogenesis. Future identification of this activity will improve our understanding of embryonic versus adult regulation of tissue regeneration suggesting novel strategies for organ rejuvenation. Comprehensively, the current intersection of aging and stem cell science indicates that if the age-imposed decline in the regenerative capacity of stem cells was understood, the debilitating lack of organ maintenance in the old could be ameliorated and perhaps, even reversed.

Mammalian aging is characterized by a myriad of changes that span many levels of complexity: from molecules to organ systems. With age, damage to DNA and proteins, misfolding, misprocessing and mistargeting of key macromolecules all increase, while the timely repair and degradation of defective components decline [1-4]. Old differentiated cells lose their functionality while stem and progenitor cells dedicated to generating new differentiated cells lose their regenerative capacity [5, 6]. The cumulative molecular and cellular changes which are characteristic of mammalian aging result in the widespread decline in organ function and the inevitable death of the organism. Such intrinsic aging seems unavoidable, since it is caused simply by utilizing (over a particular period of time) the physiological signaling pathways necessary for cell function and survival, for example, the mTOR pathway, IGF/insulin pathway or Reactive Oxygen species (ROS)-specific signal transduction [1, 7-12].

The silver lining to the gathering clouds of aging seems to be the relative youth of organ stem cells. Organ stem cells are sequestered sometime during embryonic development to remain undifferentiated yet dedicated to a particular cell lineage, and these cells reside in their tissue niches, which they maintain and repair typically throughout adult life. Organ stem cells self-renew via asymmetric cell divisions, where one daughter cell differentiates while the other remains a stem cell. The regenerative capacity of organ stem cells is very efficient in embryonic and young organisms, but deteriorates with advancing age, and the cell-extrinsic changes, i.e. the aging of the stem cell niche, play a large role in that age-specific decline. For example, it was found that satellite cells (muscle stem cells), residing in old muscle do not irreversibly lose their ability for tissue repair, and in contrast are capable of robust myogenesis when these cells are exposed to the external environments typical of a young mammal [13-18]. An undefined youthful environment, i.e. heterochronic parabiosis, where aged mice are exposed to young blood circulation, was demonstrated to enhance endogenous regeneration in muscle and liver [14, 19]. Similarly undefined, in molecular terms, transplantation of human embryonic stem cells (hESC) into the muscle of old mice rejuvenated repair after injury in vivo [20]. More defined conditions, such as activation of IGF-1 or Notch, or MAPR/pERK pathways and attenuation TGF- β signaling and Wnt signaling were reported to awaken the regenerative responses of the aged muscle stem cells [11, 13, 17-19, 21]. All these conditions are generally mitogenic, suggesting that efficient proliferation is one key component in the regenerative responses of organ stem cells. Likewise, genetic inactivation of the cyclin-dependent kinase (CDK) inhibitor, p16 was shown to rejuvenate responses of tissue dedicated stem cells in blood, brain and pancreas, while the over-expression of p16 caused premature aging of lymphocytes [22-26]. These findings suggest that aged tissue imposes a state of anti-proliferation on the organ stem cells, which then logically prevents their regenerative performance. A high threshold for stem cell activation in an old niche might be a very important adaptation as a barrier to cancer. Since DNA damage accumulates with time and translates into mutations in dividing cells, it would be beneficial to limit the process of regeneration, thus limiting the numbers of dividing stem and progenitor cells in a given tissue, because such cells could potentially deviate into neoplasty. In concert with this notion is the complex behavior of the senescent niche, in which the cell secretome changes in response to DNA damage and potentially adapts tissue to such damage. Senescent cells undergo growth arrest via cell-intrinsic and extrinsic (cytokine-imposed) mechanisms, which initially suppresses tumorogenesis and inhibits tissue regenerative responses; however, with prolonged time in senescence, some of the secreted molecules, such as IL6, IL8 and MMPs enhance tissue repair, and promote tumor progression. While the senescent niche can signal its own clearance via the immune system, some senescent cells persist and produce cytokines, causing chronic inflammation and tissue aging [27-29].

Interestingly, the expression of moderately increased levels of p19ARF/p53 cell-cycle inhibitors in transgenic mice that also over-express telomerase promoted the longevity and decreased the biomarkers of aging [30]. These results suggest that determinants of healthy and prolonged life might be under a combinatorial control of cell cycle check point proteins and mitogens, where the activities of these reciprocal regulators of cell division need to be tightly balanced.

Comprehensively, current research at the cross-roads of aging and stem cell biology hints that if the molecular changes causal to the age-imposed decline in the responses of organ stem cells were understood, the debilitating phenotypes of aging that are caused by the abandonment of tissue maintenance, could be ameliorated and perhaps, even reversed.

The key age-specific changes in signal transduction are relatively well understood in skeletal muscle: old myofibers fail to up-regulate the Notch ligand, Delta, in response to muscle damage, and in addition, overproduce TGF-β, which boosts the levels of CDK inhibitors in satellite cells thus suppressing muscle regeneration [16]. Remarkably, these biochemical features of stem cell aging are conserved between mice and humans [18]. Thus, the lessons learned in the mouse system are likely to be applicable for understanding human aging and for developing novel anti-aging clinical strategies. In human muscle it is the age-specific loss of MAPK/pERK signaling strength that precludes the up-regulation of Delta and activation of Notch in old satellite cells which consequentially fail to recognize the need to regenerate muscle [18].

The interactions between MAPK and Delta/Notch signaling are also conserved between mammals and Drosophila, where the MAPK pathway is similarly required for the induction of Delta and activation of Notch leading to increased cell proliferation [13, 18, 31, 32]. Interestingly, however, while in mice and humans active MAPK and Notch signify stem cell youth and these pathways become lacking with aging, in adult flies, the up-regulation of MAPK and active Notch actually occurs during aging and accounts for an age-specific hyper-proliferation of stem cells in the intestine [13, 18, 31].

Published results also establish that there is an age-specific increase in *systemic* TGF-β, which intriguingly is also evolutionary conserved between mice and humans [17]. These same data point out that TGF-β is likely to exert its inhibitory affects on satellite cells through the local muscle niche or when tissue is wounded and platelets release this cytokine [17]. Notably, the same work defines that Wnt, which was also reported to inhibit satellite cell myogenicity, neither increases with age systemically, nor is present in blood serum of mice and humans [17, 19]. Other systemic pro-inflammatory cytokines, such as TNF-a and IL-6, were reported to become over-pronounced in the aged mammals, and it would be interesting to determine their contribution to the decline in the regenerative responses of old organ stem cells [33].

The relative intrinsic youth of tissue stem cells in an old organism is not typical just for muscle and indeed is manifested, for example, by epidermal stem cells [34]. Similarly, the dominant age-specific role of the niche over the stem cell properties is not unique to muscle and is displayed, for example, in oogenesis [35-37]. Hence, strategies aimed to boost the performance of organ stem cells in aged tissues might succeed in general terms for rejuvenating tissue maintenance and repair throughout an old organism.

One approach for achieving the youthful responses of organ stem cells in an old mammal might enlist the help of bioengineering, for example, to generate bio-polymers that would release TGF-β antagonists, Notch and/or MAPK agonists, etc. at precise doses and rates. This is technically feasible, and biopolymers releasing cytokines, chemokines, nucleic acids, and antibodies were successfully tested in vivo for a variety of applications ranging from regenerative medicine to anti-cancer treatments [38-40]. The in vivo applications of anti-aging biomaterials would protect tissue stem cells against the aged biochemical milieu through optimizing the micro-niche and would evoke efficient regenerative responses from old stem cells, or from young stem cells transplanted into aged organs. The importance of such biomaterials is underscored by current data suggesting that even young cells are doomed to perish without a youthful niche when introduced into an old organ [20, 41]. Work in these directions are underway and the idea to provide endogenous or transplanted stem and progenitor cells with better micro-environments is being pursued by many researchers in the fields of regenerative medicine, material science and bioengineering, as well as the arena of stem cell and aging research [39,[40].

Tissue regeneration declines with aging throughout the old body, suggesting that the age-specific regulation of organ stem cells might be conserved between multiple organs. In this regard, the activation of Notch was found to be critically required not only for adult myogenesis, but also for maintenance of neural stem cells and a proper control of adult neurogenesis [13, 42]. Emerging work also suggest that the regenerative responses of adult stem cells in muscle and in brain are controlled by micro-RNAs, and that such regulation in muscle targets Pax7 and Pax3, while in brain the potential targets of micro-RNAs are the age-specific IGF and FoxO3 pathways. Future work on defining the age-imposed changes in the regulation of organ stem cells by micro-RNAs is likely to yield a better understanding of the regenerative decline in the old [43-46].

Achieving precisely “young” levels of TGF-β, Notch and MAPK signaling is critically important, because these pathways generally regulate cell growth and differentiation and control p53, p21 and other CDK inhibitors, as well as cMyc and other pro-oncogenes [47-50]. Thus, deviation from the physiologic levels of signaling strength in these pathways is very likely to cause undesired changes in multiple tissues, including but not limited to oncogenic transformation. The risk of cancers is not higher, but is typically lower in younger mammals as compared with older ones, which suggests that youthful levels of the key biochemical pathways that regulate organ stem cell responses would result in a healthier and better regenerating tissue. However young mammals also have less time to accumulate damage to DNA and other macromolecules and have high capacity to deal with such damage. Hence, even if TGF-β, Notch, MAPK, and other mitogenic pathways that regulate tissue regeneration were calibrated to their precise “young” levels in old mammals, the expectation of rejuvenated, healthy tissue and perhaps, of a longer and cancer-free life span, needs to be justified experimentally.

In practical terms, not enough is known about the identity and the expression profile of natural agonists and antagonists that modulate the signaling strength of TGF-β, Notch, MAPK and other age-specific regulators of stem cells in embryonic, versus adult and old mammals. Hence, approaches for boosting tissue repair are not based on a clear understanding of the physiologic regulation, where robust formation of embryonic tissues is followed by less efficient, but still good, tissue regeneration in adults, and deteriorates into poor tissue repair in the old. A better characterization of the transition from inductive to inhibitory modes of stem cell regulation during the progression from embryo to young, adult and aged mammal might help to uncover the key physiological molecules that modulate the age-specific rate of regeneration. Ultimately, this would lead to novel clinical applications for safely enhancing tissue regeneration in the old without the side effects associated with non-physiological disruption in signal transduction.

As an initial step in this direction, we put forward data suggesting that embryonic cells have a natural capacity to combat the inhibition of tissue regeneration via production of soluble secreted molecules and that such a youthful barrier to aging is lost when these cells differentiate.

Embryonic stem cells (ESCs) have a virtually infinite capacity to self-renew (e.g. to give rise to another embryonic stem cell), and to be pluripotent (e.g. to differentiate into virtually every cell type in the mammalian organism) [51, 52]. The tremendous potential of ESCs for organogenesis, including those of human origin (hESCs), has created great interest in understanding and deliberately controlling their cell fate determination, and tremendous progress was made in recent years in this field[53, 54]. At the same time, much less is known about the properties of self-renewing, undifferentiated hESCs that do not directly relate to developmental lineage progression, but that might indirectly influence the regenerative capacity of post-natal tissues. In this respect, our work uncovered that in co-cultures with mouse muscle precursor cells, hESCs dramatically enhanced myogenesis in vitro, and moreover, rejuvenated the repair of injured muscle in old mice when injected intramuscularly into immuno-compromised animals, even though the hES cells themselves did not contribute to new muscle tissue [20]. The same work hinted that the positive regulation of mouse myogenesis by hESCs might be due to the soluble factors (be they protein, lipid, sugar or other macromolecules), and that human mesenchymal stem cells lack this pro-regenerative activity [20]. The data and discussion below introduce evidence that self-renewing human embryonic stem cells, but not differentiated hESCs (hESCs vs. dhESCs), secrete *proteins* that counteract the oppressive biochemical milieu of aged muscle and old circulation, and restore efficient myogenesis to old satellite cells that associate with old myofibers and are exposed to old blood serum. Furthermore, we show that this embryonic activity requires an intact MAPK pathway, because the pro-regenerative effects of hESC- secreted factors become abolished in the presence of a MEK inhibitor. Interestingly, hESC-secreted factors also promote proliferation and delay differentiation of primary myoblasts, which is a typical effect of other molecules known to enhance adult myogenesis, such as Notch [13, 55, 56].

An experimental system that successfully mimics the in vivo myogenesis and is particularly suitable for comparing young and aged mammals [14, 15, 55], has been tailored here for characterizing the influence of embryonic and neonatal factors on regenerative capacity of satellite cells and for determining whether the activity is associated with proteins. Specifically, young and old satellite cells activated by muscle injury were cultured in association with their own myofibers in medium with 10% blood serum of their own age. These satellite cell cultures were maintained for 24 hours in HAM's F10 supplemented with 50% OPTI MEM (control medium), or 50% OPTI MEM-conditioned medium from hESCs or dhESCs. Exogenous MEK inhibitor was added to some samples to test the reliance of the system on MAPK pathway. BrdU was added to the cell cultures for the last 2 hours to label cells in S-phase of the cell cycle. The myogenic proliferative response of satellite cells was determined based on the percentage of proliferating myoblasts generated by the satellite cells (desmin+ve/BrdU+ve cells) [14, 16]. The enhancement of myogenic capacity was also determined by measuring continuous proliferation and delayed differentiation of myoblasts in standard differentiation promoting medium, by quantifying the percent of multi-nucleated terminally differentiated eMyHC+ myotubes vs. BrdU+ mono-nucleated cells. Typically, more than 50% of primary myoblasts fuse into post-mitotic multi-nucleated myotubes in the low mitogen differentiation medium (DMEM+2%horse serum), however, factors known to boost the regenerative capacity of muscle, such as active Notch, can delay this terminal differentiation in favour of continuous progenitor cell proliferation [55, 56].

These experiments demonstrated that secreted protein(s) in conditioned culture supernatants from hESCs manifested a pro-regenerative activity that enhanced and importantly, rejuvenated the regenerative capacity of satellite cells (Figure 1 A) and promoted the proliferation of myoblasts (Figure 1 B). Interestingly, differentiated progeny of hESCs do not posses this pro-regenerative anti-aging activity, as myogenic responses in supernatants from differentiated hESCs were no higher than that in control medium. The pro-regenerative activity was also tested and not found in embryoid bodies, suggesting that it is general differentiation and not commitment to a particular lineage that abrogates the production of the anti-aging factor(s). The pro-myogenic effects of hESC supernatants were significantly reduced upon protease treatment (Proteinase K conjugated to agarose beads following by the removal of the beads), indicating a protein source of this activity. The rapid loss of the pro-regenerative activity of hESC supernatant upon repeated freezing-thawing also indicates that the factors are labile proteins.

**Figure 1A.**
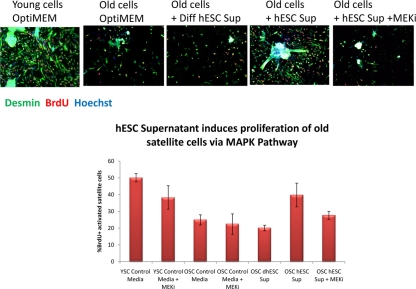
Young and old myofibers were isolated from hind leg mouse muscle at 3 days post injury by cardiotoxin and were cultured for 24 hours in Ham's F10 supplemented with 10% young or old mouse serum and 50% of the supernatant specified. 10 μM of MEK inhibitor was added to some wells, as indicated. Proliferating muscle progenitor cells that were generated by the activated satellite cells were immunodetected with anti-desmin (green) and anti-BrdU (red) antibodies; Hoechst (blue) was used to label all nuclei. Percent of proliferating myogenic cells was determined by CellProfiler. Typically poor myogenicity of old satellite cells cultured with old serum was rescued by hESC supernatant in a MAPK-dependent manner.

**Figure 1B.**
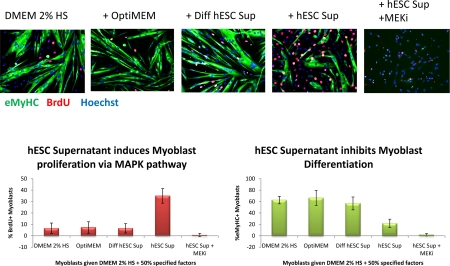
Primary myoblasts were cultured for 24 hours in DMEM + 2% Horse Serum and 50% of the supernatant specified. 10 μM of MEK inhibitor was added to some wells, as indicated. At 24 hours, cells were pulsed with 10 μM BrdU for 2 hours and fixed with 70% ethanol. Cells were immuno-stained for eMyHC (green) and BrdU (red); Hoechst (blue) was used to label all nuclei Automated imaging of these cells was done using ImageXpress and automated counting of percent of eMyHC+ and BrdU+ cells was performed by quantifying at least 100 sites per experimental sample by MetaExpress. hESC supernatant enhanced myoblast proliferation in a MAPK-dependent manner and diminished differentiation into myotubes.

Quite interestingly, the pro-regenerative activity was found to be dependent on intact MAPK signalling as there was no enhancement of myogenesis in the presence of a MEK inhibitor (Figure 1 A and B). For satellite cells, inhibition of the MAPK pathway reduced the pro-regenerative effects of hESC supernatants on old satellite cells and slightly diminished the proliferation of young satellite cells (Figure 1A). The attenuation of satellite cell proliferation by MEK inhibitor was partial suggesting that additional positive regulators of cell proliferation (for example, active Notch) might play a role in the studied experimental system. For myoblasts, the inhibition of MAPK signalling prevented both proliferation and differentiation of the majority (~88%) of cells cultured with hESC supernatants (Figure 1 B), while a few cells (~12%) differentiated into multinucleated myotubes with BrdU-low nuclei. It has been shown that MAPK signalling is important for the G1 to S transition, but once cells enter S phase, they complete cell cycle independently of this pathway [57]. Hence, it is possible that some myoblasts in the G1 phase of the cell cycle failed to enter the S phase in the presence of MEK inhibitor, even though the hESC supernatant was present, while myoblasts that were already in S or G2/M phases completed the cell cycle and differentiated into myotubes instead of entering into another G1 phase. While two different phenotypes were observed, in both cases the hESC-derived pro-regenerative factors were not capable to promote proliferation or delay differentiation of primary myoblasts when the MAPK pathway was experimenttally inactivated.

Summarily, these results suggest that self-renewing human embryonic stem cells, but not their immediately differentiated progeny, produce soluble pro-regenerative protein(s) with anti-aging activity, which require an intact MAPK pathway for their positive effects on adult myogenesis. The identification of the pro-regenerative protein(s) is to follow, and the reliance of adult myogenesis on MAPK signalling suggest some interesting candidate gene approaches for uncovering natural molecules that counter the effects of aged niches on organ stem cells. Molecular identification of this activity will broaden our knowledge of embryonic, adult and aged regulation of tissue regeneration and will point toward novel clinical strategies for organ rejuvenation and for improving outcomes of degenerative disorders where endogenous progenitor cells are overwhelmed by continuous tissue death. Since the activity is produced by human cells and manifests in mouse cells, it is likely that the secreted proteins are evolutionarily conserved.

That is interesting from a theoretical standpoint and provides a tractable experimental system for addressing the abovementioned questions.

Interesting recent work suggests that a number of micro-RNAs attenuate the levels of SIRT-1 (a factor broadly implicated in senescence and aging), when mouse embryonic stem cells differentiate or upon a transition from embryonic to adult cells [58, 59]. The genetic and epigenetic changes between an embryonic versus an adult mode of tissue maintenance are likely to be numerous and some might explain the diminished, and eventually lacking, capacity for regeneration that is typical of human aging.

While it is generally believed that embryonic stem cells do not exist postnatally, a very interesting recent study reports the presence of pluripotent Oct-4+ SSEA-1+Sca-1+Lin-CD45- very small embryonic-like stem cells residing in the adult murine bone marrow and other tissues [60]. Furthermore, it is suggested that the numbers of these cells, and thus the “youthful” tissue regenerative capacity, are under the negative regulation of Insulin/IGF, which fits with many studies showing an increase in longevity of animals that are deficient in Insulin/IGF [60-62]. Future work is needed to determine whether these embryonic-like pluripotent cells physiologically differentiate into various tissues in adults and whether there is an age-specific decline in this mode of postnatal organogenesis.

In conclusion, the negative influence of aged differentiated niches (both local and systemic), on organ stem cell responses becomes pronounced in the old. Additionally, the mechanisms ensuring high performance of embryonic stem cells are tuned down or switched off later in life. One reason for such an attenuation of stem cell responses might be that the risk of cancer poses a greater threat to longevity as compared to the risk of diminished tissue repair. In other words, if tissue regenerative capacity becomes diminished with age, there are fewer chances for organ stem and progenitor cells to deviate from their lineage commitment and generate tumors. From this perspective, the age-specific decline in mitogenic signals, such as the embryonic pro-regenerative activity or active Notch and MAPK pathways, compounded by a rise in TGF-β and CDK inhibitors, might have evolved to serve a protective role, where the fitness of a mammal is actually increased even though responses of muscle stem cells (and perhaps, other organ stem cells), are restricted. The progress of current research is rapidly improving our understanding of the properties of embryonic, adult and aged stem cells, and will certainly provide a better definition of the molecular determinants of tissue repair and maintenance. Ultimately, new therapies for combating and reversing tissue aging will be developed.
